# Co-Targeting the Dysregulated Ocular and Cochlear Blood Flow via Ophthalmic Nerve Stimulation for the Treatment of Type 2 Usher Syndrome: Prospective Case Series

**DOI:** 10.3390/jcm15218302

**Published:** 2026-10-25

**Authors:** Ismail M. Musallam

**Affiliations:** Musallam Eye Center of Jerusalem, Beit Hanina, Baldi Mall, B Building, Jerusalem 9160201, Israel; musallamcenter.jerusalem@gmail.com

**Keywords:** Usher syndrome, retinitis pigmentosa, ocular neuromodulation, ophthalmic nerve stimulation, Low luminance questionnaire-10, rod responders, ascorbic acid, substance P

## Abstract

**Background/Objectives**: Dysregulated ocular and cochlear blood flow are proposed pathways in the pathogenesis of type 2 Usher syndrome (USH2). This study aimed to evaluate the safety and preliminary efficacy of ophthalmic nerve stimulation (ONS) combined with intravenous ascorbic acid (AA) in patients with USH2. **Methods**: Nine participants with USH2 were enrolled in a prospective, open-label, interventional case series. All participants received daily ONS combined with intravenous AA over a two-week period. The primary endpoint was the 6-month change in self-reported scotopic function, assessed using the 10-item Low luminance questionnaire (LLQ-10; 0–100 scale). Responders were defined as participants demonstrating a ≥25-point increase in LLQ-10 score. Secondary outcomes included changes in best-corrected visual acuity (BCVA), contrast sensitivity (CS), and subjective hearing function. **Results**: ONS-based therapy resulted in a significant improvement in LLQ-10 scotopic scores at 6 months (mean change: +42.1 ± 11.3 points, *p* = 0.0001), with seven of nine participants (77.8%) classified as responders. Clinically meaningful improvements in BCVA (≥0.2 logMAR) and CS (≥0.3 log units) were observed in 22.2% of tested eyes. Additionally, subjective hearing improvement was reported by one-third (33.3%) of participants. No severe adverse events were reported. **Conclusions**: ONS combined with AA demonstrated safety and yielded significant subjective improvements in night vision quality among patients with USH2, alongside minor improvements in visual acuity, contrast sensitivity, and hearing in a subset of participants. Controlled trials incorporating objective scotopic and audiometric testing are warranted.

## 1. Introduction

Usher syndrome (USH) represents a group of autosomal recessive disorders characterized by sensorineural hearing loss (SNHL), vision loss, and vestibular dysfunction [1]. It primarily affects the auditory hair cells followed by light-sensitive photoreceptor cells in the retina [2]. USH is the leading genetic cause of deaf–blindness (50%of all cases), with a prevalence in the range of 4 to 17 per 100,000 people [3].

Three clinical subtypes of USH have been defined—Type 1, Type 2, and Type 3—based upon the severity and progression of SNHL. Type 2 (USH2) is the most common subtype, accounting for more than half of all USH cases worldwide [4]. USH2 patients present with moderate-to-severe SNHL, normal vestibular function, and retinitis pigmentosa (RP) that begins generally during puberty.

There is no cure for USH2. Several treatment options are available to help patients manage hearing problems. This includes auditory rehabilitation using hearing aid devices or cochlear implants. Unfortunately, these technologies do not treat the underlying cause of hearing loss and are far from providing quality natural hearing. Moreover, there is no cure for RP. Currently, there are a range of new therapeutic strategies under investigation that utilize new technologies such as gene and cell-based therapies. Given that USH2 is a syndromic condition affecting multiple senses, it is important to note that current therapeutic trials generally focus on one sense rather than the full spectrum of the disease. Therefore, a comprehensive therapeutic strategy is needed that simultaneously targets the underlying pathophysiology in both the retina and the inner ear regardless of the genetic background of the disease.

The human inner ear depends on a vascular supply to maintain fluid homeostasis, ion balance, and metabolic supply. Disruption of cochlear blood flow (CoBF) leads to instantaneous pathological changes in the inner ear [5,6]. A growing body of evidence suggests that microvascular changes may contribute to hearing loss [7,8,9,10,11]. Histopathological examination of the temporal bone of patients with USH revealed atrophy of the stria vascularis [12]. This is thought to cause the degeneration of hair cells by alteration of the endolymph composition [13]. The stria vascularis, a highly vascularized region located in the cochlear microcirculation, is the metabolic energy source underlying cochlear function [14].

Circulatory changes have also been observed in the retina and choroid of patients with Usher’s syndrome. The vascular density was significantly decreased in the retinal circulation of patients with *USH2A* and *MYO7A* mutations compared with controls [15]. Changes were observed in both the superficial and deep capillary plexus. The vascular density in the deep capillary plexus showed a strong association with macular sensitivity and the ellipsoid zoon width [15]. Choroidal thickness is reduced in people with USH2 compared to healthy subjects., which might be considered a sign of blood flow reduction [16]. Evaluating choroidal hemodynamic changes has shown that the reduction in choroidal blood flow (ChBF) is proportional to the progression of RP [17]. Recently, relative choroidal ischemia and reduced ChBF have been demonstrated in RP by measuring the choroidal vascularity index via SD-OCT [18]. The ocular blood flow (OBF) is reduced not only in the retina and choroid but also in the retro-ocular vessels [19].

The eye receives its blood supply through anterior cerebral circulation (internal carotid arteries) via ophthalmic arteries. The retina has a dual blood supply, with the retinal circulation supplying the inner layers and the choroidal circulation supplying the outer layers of the retina including photoreceptors. The blood supply to the choroid in mammals arises from the ophthalmic artery via the long and short ciliary arteries. Like the eye, the inner ear is supplied with an end artery. The blood supply to the inner ear is derived from the posterior cerebral circulation (basilar artery), mostly from the labyrinthine artery, a branch of the anterior inferior cerebellar artery.

The ophthalmic artery, central retinal artery, posterior ciliary arteries, and choroid are richly innervated by parasympathetic, sympathetic and trigeminal sensory nerve fibers. Parasympathetic innervation has been shown to cause vasodilation and increase ChBF, and sympathetic input has been shown to induce vasoconstriction and decrease ChBF. In contrast, autonomic control for cochlear blood vessels is limited to sympathetic innervation that arises from the superior cervical ganglion, whereas parasympathetic innervation of cochlear blood vessels has not been found [20].

The majority of the intracranial blood vessels including those contributing to both the ocular and cochlear circulation are innervated by the ophthalmic division of the trigeminal nerve [20,21]. Direct stimulation of the trigeminal ganglion in experimental models has been found to lead to increased cerebral blood flow [22]. Stimulation of the ophthalmic nerve may be topographically oriented, with specific branches leading to increased blood flow in specific regions of the eye, ear, and brain arterial tree.

The ophthalmic nerve plays a pivotal role in the regulation of ocular and CoBF (Figure 1) by inducing vasodilation, as a result of the release of substance P (SP), calcitonin gene-related peptide (CGRP), and neuropeptide Y as mediators of vasodilative mechanisms [23,24]. Stimulation of sensory afferents from the ophthalmic nerve results in the activation of the trigeminovascular system via antidromic impulse [25,26,27]. The nasociliary nerve, which originates from the ophthalmic nerve, contains major vasodilatory innervation [28,29], and its stimulation results in the release of vasoactive neuropeptides from the free nerve endings, such as SP and CGRP [23,24]. Consistent with this, stimulation of the ophthalmic nerve in experimental animals was found to increase OBF [30], decrease carotid arterial resistance, [31] and enhance the uveal release of SP [32]. Infusion of the internal carotid artery or the anterior inferior cerebellar artery with SP induces an increase in CoBF [33].

There is extensive evidence that excessive free radical formation along with diminished CoBF are essential factors involved in the pathogenesis of SNHL [34,35]. Antioxidant interventions have been reported to substantially delay the death of photoreceptor cells of the retina [36] and hair cells of the inner ear [37]. Interestingly, a high dose of vitamin C has been shown to enhance hearing recovery in idiopathic sudden SNHL patients, which suggests that ascorbate treatment reduces levels of reactive oxygen metabolites produced by inner ear ischemia or inflammation [37]. Therefore, it is reasonable to suggest that supra-physiological doses of intravenous AA could be beneficial in a perfusion-compromised retina or inner ear, as in USH2. Preclinical studies show that a high dose of AA can prevent or restore microcirculatory flow impairment. AA can restore vascular responsiveness to vasoconstrictors and preserve the endothelial barrier [38]. Additionally, AA exerts sympatholytic effects via central action [39].

The pathogenesis of retinal and inner ear disorders in USH2 reflects a summation of primary reduced dysregulated OBF/CoBF and consecutive unstable oxygen supply, hair cell/photoreceptors starvation, oxidative stress and inflammation of the retina and the inner ear. These vascular and cellular processes are ultimately combined to produce damage of cochlear hair cells followed by retinal rods and finally cone photoreceptors apoptosis. The mechanisms responsible for reduced dysregulated ocular and CoBF in patients with USH2 remain uncertain. It is also uncertain whether blood flow decline stems from disturbed neural control of ocular and cochlear circulation or vascular pathology or both.

The reduced dysregulated CoBF/OBF is proposed as a common pathway in the pathogenesis of SNHL and blindness, which can be a target for a novel therapeutic strategy in USH2. Because of the complexity of this syndrome, it is unlikely that optimal protection of the inner ear mechanoreceptor and retinal photoreceptors can be achieved through a single therapy, suggesting that an alternative approach based on combination therapy to be considered. Upregulation of the trigeminovascular system and parasympathetic system via ONS along with antioxidation, sympathetic inhibition and restoration of vascular endothelial function via systemic administration of AA might form the basis of neuromodulation therapy for treating this non-curable multi-sense damaging syndrome. Here, we report the safety and efficacy results of ONS paired with systemic ascorbate for treating participants with USH2.

## 2. Materials and Methods

The study was a prospective, consecutive, open-label, interventional study aimed at investigating the safety and efficacy of ONS combined with AA on patients with USH2. The protocol was reviewed and approved by the Ethics Committee of the Musallam Specialty Hospital, Palestinian Authority, and it adhered to the tenets of the Declaration of Helsinki. Written informed consent was obtained from all participants or legal guardians after an explanation of the purpose and possible outcome of therapy.

### 2.1. Patient Selection

Patient enrollment started in April 2018, and cases were recruited from a single tertiary ophthalmic center. The inclusion criteria of the study included patients with an established diagnosis of USH2 with BCVA ≥ 20/400. The exclusion criteria were pregnancy, glaucoma, CRVO and retinal detachment. bradycardia, malignancy, and active infection.

Following recruitment, all participants with USH2 completed baseline vision testing, slit lamp examination and fundoscopy. Spectral Domain Optical coherence tomography (SD-OCT) was performed within 2–4 weeks prior to treatment. The severity of the RP associated with Usher’s syndrome was clinically graded into six stages based on fundoscopy and SD-OCT findings [40].

The primary efficiency endpoints were 6-month changes in scotopic vision and adverse effects of the therapy. The scotopic vision was assessed with a Low luminance questionnaire-10 (LLQ-10) [40]. The secondary efficiency points include the best-corrected visual acuity (BCVA), contrast sensitivity (CS), and the change in hearing acuity. BCVA was recorded in Snellen equivalents and then transformed to logMAR unit. CS was measured using a Pelli–Robson chart under standardized photopic illumination at one meter in each eye, using best correction spectacles. To characterize the severity of hearing loss at baseline, a subjective numerical rating scale (NRS) of 1 (normal hearing) to 6 (complete deafness) was used [41]. Improvement in an NRS score by 2 or more points was considered a clinically significant improvement in hearing.

To evaluate the therapy outcome, LLQ-10 score changes from baseline to 6 months after treatment were calculated (the primary efficacy endpoint). LLQ-10 has been used to quantify the participant’s visual dysfunction under low luminance, integrating aspects of visual acuity, mobility, visual field, depth of perception, reading, color vision, dependency on others’ help, social functions, and mental health [40]. Patient-centered outcome measures have been increasingly viewed as necessary in clinical trials, serving as primary endpoints [40,42,43,44]. Participants rated how much difficulty they had performing each of the activities under low luminance on a 5-point Likert scale. Rod responders were identified as those in whom the LLQ-10 score increased by ≥25 points at 6 months’ post-treatment.

For safety purposes, the vital signs and ECG were monitored before, during, and after each session of neuromodulation. Ocular and non-ocular adverse events were also reported (primary safety endpoint).

### 2.2. The Treatment Protocol

ONS-based therapy protocol included the administration of AA followed by ONS over a period of two weeks. AA was given intravenously in a dose of 3 gm dissolved in 100 mL of saline infused at 5 mL min^−1^ for 20 min on the first day, followed by a drip infusion of 1 g daily during the rest of the treatment period.

A modulated low magnitude low-frequency vibration with a frequency of approximately 60–90 Hz and stimulation amplitude range of 1.5–3.5 μm was used for ONS. Modulated frequency and amplitude waveforms were used to avoid adaptation to mechanical stimulus. A head-mounted prototype of ophthalmic nerve stimulators was applied to different application sites by using modified commercially available micro-vibrators, along with different types of intranasal and extra-nasal application heads.

Bilateral ONS was applied to the subjects over a session of 30 min per day, 6 sessions per week, and one day off. Each session included intra-nasal vibrochemical and extra-nasal vibrotactile stimulation over the nasal bridge and the supraorbital region in both sides, using appropriate application heads. During intra-nasal vibrochemical stimulation, the nasal application head was covered by a rubber cap impregnated by 2% menthol cream (Dermacool plus^®^; Dermal Laboratories Ltd., Hitchin, Hertfordshire, United Kingdom) as a transient receptor potential cation channel subfamily M (melastatin) member 8 (TRPM8) agonist. Participants were assessed clinically and by SD-OCT and ILQ-10 at baseline and at 1 and 6 months after treatment.

### 2.3. Statistical Analysis

The statistical analysis was performed using SPSS^®^, V. 21. The change in the mean value of visual function parameters between baseline and different post-treatment points was evaluated using a paired sample *t*-test. The BCVA and contrast sensitivity at baseline and at post-treatment visits were recorded for the left eye in the whole cohort. All tests were two-tailed, and a *p*-value less than 0.05 was considered significant.

The proportion of left eyes that showed a clinically significant improvement in visual function among the whole cohort was also evaluated. Additionally, the proportion of patients who experienced a clinically significant improvement in hearing acuity was also determined.

## 3. Results

### Baseline Demographics

Between 14 April 2018 and 29 April 2020, nine participants, aged between 8 and 55 years (median: 23 years, mean: 24.8 ± 13.9), with seven females (77.8%) were recruited. The demographics and baseline characteristics of the participants are presented in Table 1.

At the 6 month visit, seven out of nine (77.7%) patients were defined as rod responders. In the whole cohort, the night vision score was improved from 21.4 ± 11.3 points at baseline to a level of 59.2 ± 19.8 points (*p* = 0.0001) at 1 month and 56.4 ± 18.7 points (*p* = 0.0001) at 6 months (Figure 2), with an overall improvement of (35.0 ± 17.2 points) as compared to the baseline (*p* < 0.0001). The mean change in LLQ-10 score was 45.4 ± 8.1 points (*p* = 0.0001) at one month and 42.1 ± 11.3 points (*p* = 0.0001) at 6 months in rod responders. The two non-responders have stage III and V RP, and the duration of night blindness was 12 and 20 years (16 ± 5.7).

The BCVA of the LE was improved from (0.38 ± 0.43 logMAR) at baseline to (0.26 ± 0.33 logMAR) (*p* = 0.19) at 1 month and (0.28 ± 0.34 logMAR) (*p* = 0.24) at 6 months, which was found statistically not significant. In two out of nine patients (22.2%), a clinically significant improvement in BCVA (improvement of ≥0.2 logMAR) was noticed.

Regarding CS, the mean CS of the left eye was improved from 0.64 ± 0.60 log unit to 0.79 ± 0.57 log unit at 1 month and 0.76 ± 0.54 log unit at 6 months. However, this change was not statistically significant. At the 6 month visit, two eyes (22.2%) in the whole cohort had a clinically significant improvement in CS (improvement of ≥0.3 log unit).

Typically, NSHL is the first symptom in the natural history of USH2. No change in hearing was expected after treatment, and thus it was surprising to observe that a clinically significant improvement in hearing was observed in three patients (33.3%) during the study period. This improvement in hearing in these patients are unlikely to be due to chance.

No serious adverse effects were reported, and headache in one patient was the only encountered side effect in this study.

## 4. Discussion

To date, there is no treatment option available to cure sensorineural hearing loss or vision loss in USH2, and only prostheses such as hearing aids and cochlear implants offer some help. In the current study, we have the reported successful use of ONS-based therapy as a novel treatment for USH2 syndrome. It has led to a clinically meaningful and statistically significant improvement in scotopic vision. Additionally, a clinically significant improvement in BCVA, CS and hearing was also noticed in some patients.

In this study, we found that the most robust improvement in visual function for the subjects with USH2 was night vision as measured by the LLQ-10. The recovery of night vision was likely to be related to the reactivation of dormant/starving rods via enhanced oxygen and glucose delivery and shunting metabolites toward aerobic glucose metabolism [45].

There are four clinico-pathological overlapping phases that characterize USH2: an early hair cell degeneration phase, rod degeneration phase, transitional phase of rod/cone photoreceptors damage and late cone degeneration phase. SNHL occurs prior to visual symptoms in all subtypes of USH syndrome including USH2 [2], probably because the inner ear is more vulnerable to hypoperfusion. This might be explained at least in part by the early development of dysregulated CoBF as a result of dominant sympathetic innervation of cochlear blood vessels and the absence of parasympathetic input [20]. Interestingly, cochlear circulation is innervated by branches of ophthalmic nerves [20] that produce the robust dilatation of labyrinthine arteries and their branches, increasing CoBF, as result of release of vasoactive neuropeptides. Therefore, activation of the ophthalmic nerve may counteract the dysregulated CoBF induced by increased sympathetic tone.

The vascular alteration in the inner ear and the retina might explain the sequence of pathological events in USH2. A highly vulnerable cochlear circulation compared to ocular circulation might explain the early development of SNHL. Additionally, the atrophy of the stria vascularis [12] supports the notion that auditory hair cell damage is primarily of vascular origin. Stria vascularis is a key structure for producing and maintaining the endocochlear potential. Morphologic alterations in the stria vascularis decrease the endocochlear potential and consequently affect the cochlear amplification of acoustic signals leading to an increase in auditory thresholds, even in the absence of hair cell death [34]. On the other hand, the development of annular scotoma at the mid-periphery opposite the choroidal watershed zone between the anterior and posterior ciliary arteries [46] provides further support to the vascular theory of USH2 regardless of the genetic background. SNHL including Meiners disease, sudden SNHL, senile deafness and inherited NSHL were attributed at least in part to underlying vascular causes and reduced CoBF. Nevertheless, the unmet oxygen and glucose requirements of mutant hair cells and rod photoreceptors might be detrimental for the disease process and its sequence of occurrence in USH2.

A key finding in the present study was the substantial improvement in hearing in three of the participants. Such an improvement is unlikely to be due to chance and mostly related to the effect of treatment, as this phenomenon is not normally seen in untreated patients with USH2. Hearing improvement might be related to one of the following mechanisms, including recovery of dormant hair cells, regeneration of hair cells, or trans differentiation of supporting cells into hair cells. It is reasonable to suggest that the SNHL seen in the early stages of USH2 is explained, at least in part, by hair cell dormancy and that increased CoBF and glucose delivery to the inner ear in response to ONS might restore the function of these dormant hair cells, resulting in improved hearing. Hair cell dormancy has been demonstrated in organ culture. On exposure to a low dose of the aminoglycoside antibiotic gentamicin trauma, some of the hair cells completely lose their bundles [47]. These bundleless hair cells descend towards the basal lamina, and the apical part of these mechanosensory cells become surrounded by adjacent supporting cells. These hair cells are viable cells (dormant hair cells) that can grow back to the apical surface of the epithelium, resynthesize their bundles, and thereby restore their function [47].

Although the exact mechanism of action of ONS-based therapy in USH2 is not fully understood, the distinctive effect of ONS-based therapy could be due to its capability to target diverse pathophysiologies of USH2. It is known that antidromic activation of the ophthalmic nerve, especially its nasociliary branch, leads to the release of neuropeptides such as SP from nerve terminals. SP, an 11-amino acid neuropeptide that belongs to the tachykinin family of peptides, has a preferential affinity to the neurokinin-1 receptor (NK-1R) [48]. Several studies have indicated that SP is capable of boosting both the ocular/CoBF [25,26,30,33] and tissue regeneration by the recruitment of endogenous stem cells [49]. Moreover, SP can protect diverse types of cells including retinal pigment epithelium [50] and ganglion cells [51]. It also contributes to the prevention of apoptosis [52,53] and the suppression of both inflammation [54,55] and oxidative stress [56,57]. SP was reported to modulate Akt/GSK-3β signaling, inhibit reactive oxygen species-induced cell death, preserve cell viability, and block cellular alterations [56,57,58]. SP exhibited a protective effect on the cochlea from noise damage in guinea pigs exposed to noise after an infusion of SP into the inner ear [59]. However, the therapeutic applications of SP have been limited by its low stability. SP tends to be degraded by various proteases, including neutral endopeptidase and angiotensin-converting enzymes [60,61,62]. The half-life time of SP is very short, from seconds to minutes [63,64,65].

If endogenous SP is to be therapeutically effective, then the sustained release of this neuropeptide from the nerve terminals of the ophthalmic nerve is essential, and its stability should be enhanced. SP has been identified at several locations in the auditory pathway [66,67], including the inner ear [68,69,70]. Immunohistochemical and electrophysiological studies confirm that SP may represent a neuromodulator function at the synapses of the inner hair cells in the guinea pig [71]. SP immunolabeling has been observed on the labyrinthine and spiral modiolar arteries but not in the stria vascularis [68,72]. Similarly, SP is located primarily in sensory nerves, surrounding the ophthalmic artery and its branches. The perivascular location of SP in the ocular and inner ear blood vessels makes the sustained release of this potent vasoactive neuropeptide by upregulation of the trigeminovascular system, an innovative therapeutic strategy for USH2. Concomitant infusion of AA and administration of ONS might be significantly correlated with the clinical outcome of this therapeutic strategy. AA is an efficient inhibitor for both angiotensin-converting enzyme and neutral endopeptidase enzyme, enzymes that are commonly involved in SP degradation [73,74,75].

The underlying mechanisms of retinal degeneration and hair cell loss in USH are highly complex, and oxidative stress and dysregulated blood flow may be common to retinal degeneration as well as to hair cell damage. Previous studies have demonstrated that an excess of free radical formation and blood reduction in the cochlea occur in noise, drug-induced hearing loss, and senile SNHL [35]. Then, it is reasonable to suggest that dysregulated CoBF and oxidative stress may be critical factors in triggering hearing loss associated with USH2. The production of oxidative stress via free radical generation has been implicated by a variety of insults that can result in SNHL [76]. The role of antioxidants in the prevention of hearing loss has been reported in a number of studies [37,77,78]. Interestingly, high doses of vitamin C have been shown to enhance hearing recovery in idiopathic sudden SNHL patients, which suggests that vitamin C reduces levels of reactive oxygen metabolites produced by inner ear ischemia or inflammation. Therefore, it is reasonable to suggest that a high dose of intravenous AA could be beneficial in Usher syndrome.

On the other hand, photoreceptor cells have a rich complement of mitochondria to accommodate the high metabolic rate and are more sensitive to oxidative stress than other cell types [79]. The development of RP, for instance, is tightly linked to oxidative damage [80], and antioxidant therapy can delay cell loss in retinal degenerative diseases and lead to reduced photoreceptor cell death in experimental animals [81] and in clinical trials [36]. It has been demonstrated that SP could stimulate the recovery of RPE cells under oxidative stress, possibly by promoting cell proliferation and inhibiting apoptosis through the activation of Akt/GSK-3β signaling [56].

The interpretation of these study findings should be considered within the context of a few limitations. These include the small number of participants, the short duration of follow up, the lack of information regarding the genotype, the electrophysiology of the retina, and objective hearing tests. Nevertheless, this is an open-labeled single-armed intervention and was intended to be an exploratory one. In the future, a proper control group should be considered in a large multicenter double-blind prospective study with stratified randomization.

In conclusion, this study introduces ONS-based therapy as a novel method for the treatment of USH2, a disease that currently has no treatment. It demonstrates the efficacy and safety of ONS-based therapy as a comprehensive treatment for various sense organs involved in this syndrome irrespective of genetic background. The neuroprotective effects of ONS-based therapy are probably mediated by modification of the neural circuit of ocular and cochlear circulation. Moreover, auditory hair cells and retinal photoreceptor death could be prevented by strengthening endogenous pro-survival mechanisms or by directly blocking cell death via the expression of endogenous SP by noninvasive method of ONS. Additionally, the oxidative damage for hair cells and photoreceptors can be minimized by use of supraphysiological doses of intravenous ascorbate.

## Figures and Tables

**Figure 1:**
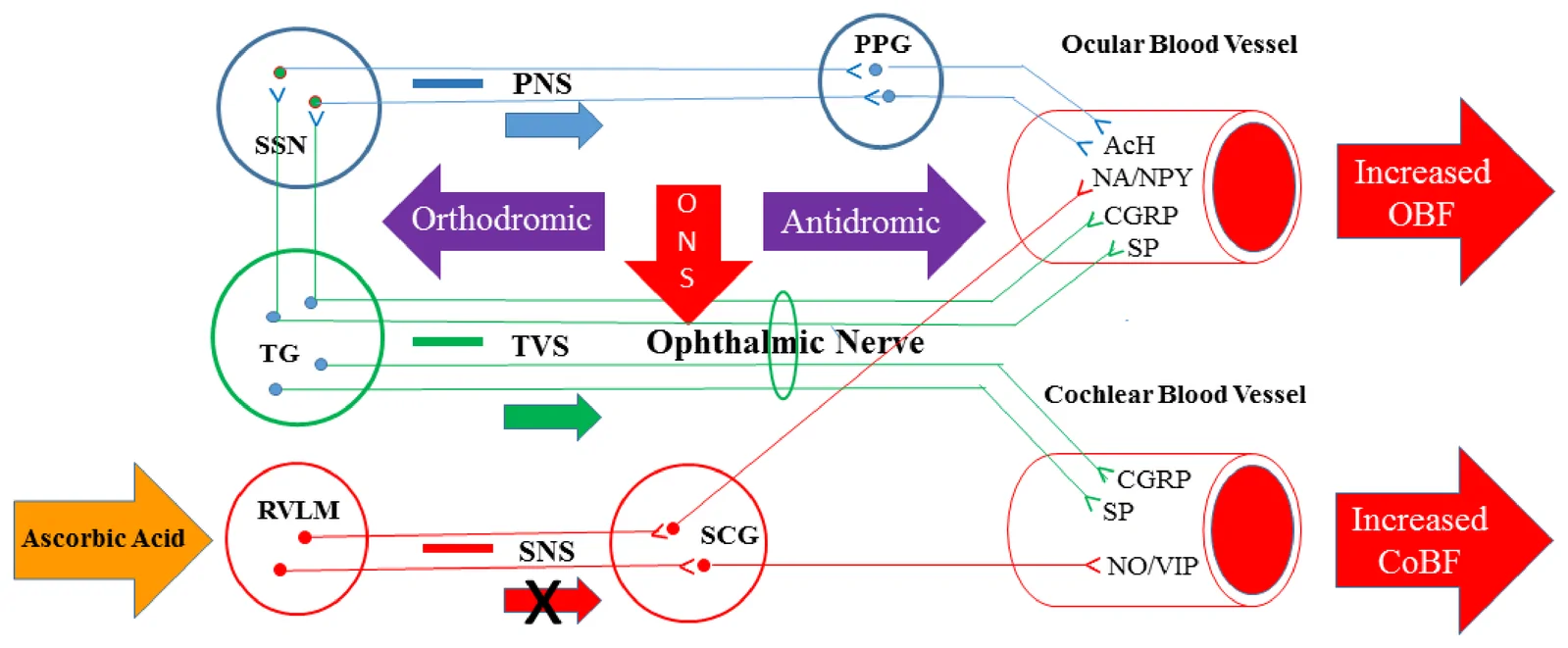
Schematic of neural pathways that control ocular (OBF) and cochlear blood flow (CoBF). ONS; ophthalmic nerve stimulation, SNS; sympathetic nervous system (Red lines), PNS; parasympathetic nervous system (Blue lines), TVS: trigeminovascular system (Green lines), TG: trigeminal ganglion, PPG: pterygopalatine ganglion, SSN: superior salivatory nucleus, SCG: superior cervical ganglion, RVLM: rostro-ventrolateral medulla, SP: substance P, CGRP; calcitonin gene-related peptide, Ach: acetylcholine, NO: nitric oxide, VAP: vasoactive intestinal polypeptide, NA: noradrenaline, NPY; neuropeptide Y.

**Figure 2:**
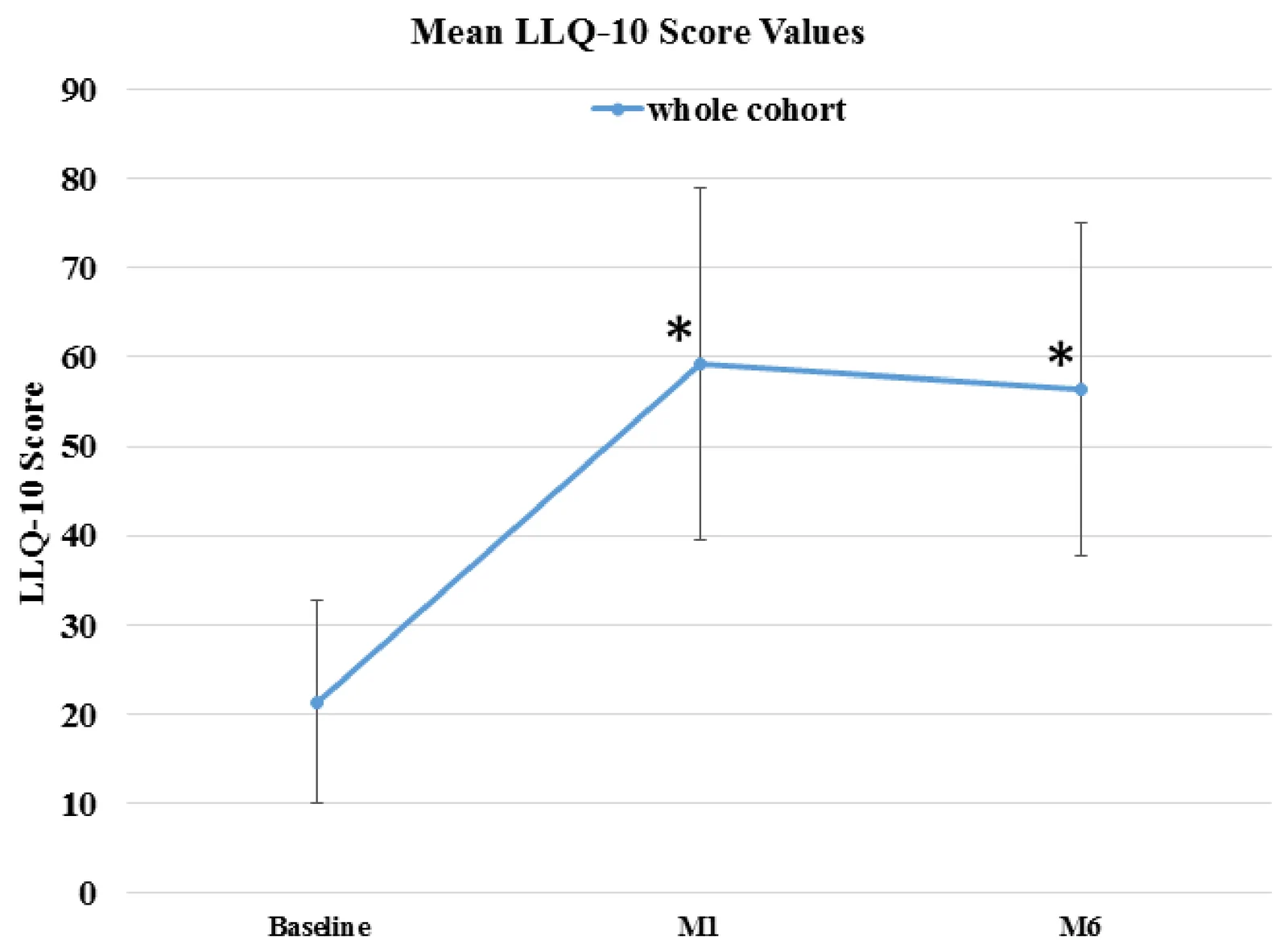
Changes in scotopic vision; mean value and standard deviation of LLQ-10 score among the whole cohort at baseline and at post-treatment visits. (* *p* ≤ 0.05); statistically significant differences in the mean value of ILQ-10 score at different post-treatment visits compared to baseline respectively.

**Table 1:** Baseline characteristics.

Baseline Characteristics	Total Cohort (9 Patients; 9 Eyes)
**Age (years), Mean ± SD**	24.8 ± 13.9
**Age Rang (years)**	8–55
**Median age (years)**	23
**Sex (Male/Female), (Male %)**	2/7 (22.2%)
**Rang of night blindness (years)**	5–20
**Duration of night blindness (years), Mean ± SD**	11.8 ± 6.2
**Duration of night blindness ≤ 10 years, n (%)**	4 (44.4%)
Stage of the Disease, n (%) *	
**Stage II**	4 (44.4%)
**Stage III**	2 (22.2%)
**Stage IV**	2 (22.2%)
**Stage V**	1 (11.1%)
**LLQ-10 Score (mean ± SD)**	22.8 ± 12.4

* Disease stages were classified based on standard clinical/funduscopic severity criteria for retinitis pigmentosa as described by Musallam IM (2025) [40].

## Data Availability

The datasets used and/or analyzed during the current study are available from the corresponding author upon reasonable request.
